# Longitudinal Changes in Nutritional, Inflammatory, and CT-Derived Body Composition Markers During Adjuvant Chemotherapy for Stage II/III Colorectal Cancer

**DOI:** 10.3390/cancers18172886

**Published:** 2026-09-06

**Authors:** Makoto Hasegawa, Tomoyuki Momma, Shizuka Kimura, Hitomi Ichinose, Hiroki Yago, Takahiro Sato, Misato Ito, Takuro Matsumoto, Daisuke Ujiie, Shun Chida, Hirokazu Okayama, Motonobu Saito, Wataru Sakamoto, Koji Kono

**Affiliations:** 1 Department of Gastrointestinal Tract Surgery, Fukushima Medical University School of Medicine, Fukushima 960-1295, Japan; 2 Department of Gastroenterology, Kita Fukushima Medical Center, Date 960-0502, Japan; 3 Department of Nutrition, Fukushima Medical University Hospital, Fukushima 960-1295, Japan; 4 Department of Gastroenterological Surgery, Aizu Medical Center, Fukushima Medical University, Aizuwakamatsu 969-3492, Japan

**Keywords:** colorectal cancer, adjuvant chemotherapy, nutritional assessment, body composition, myosteatosis

## Abstract

**Background/Objectives**: Evidence on longitudinal changes in nutrition, inflammation, and body composition during adjuvant chemotherapy for Stage II/III colorectal cancer (CRC) remains limited. We therefore evaluated these changes and explored preoperative factors associated with treatment discontinuation and prognosis. **Methods**: This single-center retrospective study included patients with Stage II/III CRC who underwent curative surgery and adjuvant chemotherapy. Body mass index (BMI), computed tomography (CT)-derived psoas muscle index (PMI), psoas muscle density (PMD), modified intramuscular adipose tissue content (mIMAC), Geriatric Nutritional Risk Index (GNRI), Prognostic Nutritional Index (PNI), hemoglobin–albumin–lymphocyte–platelet (HALP) score, neutrophil-to-lymphocyte ratio (NLR), and platelet-to-lymphocyte ratio (PLR) were assessed preoperatively and 6 months after chemotherapy initiation. Analyses were stratified into oxaliplatin-based doublet therapy (CAPOX/FOLFOX) and fluoropyrimidine monotherapy. **Results**: Among 142 patients, GNRI, PNI, and HALP score increased, whereas BMI, NLR, and PLR decreased from preoperative assessment to follow-up. Adjuvant therapy was discontinued in 45 patients (31.7%). In the doublet therapy group, discontinuation was associated with lower preoperative GNRI, PNI, and HALP scores and higher NLR and PLR. In the monotherapy group, only older age was associated with discontinuation. Lower preoperative PMD and mIMAC, but not PMI, were associated with poorer overall survival. No other nutritional or inflammatory markers were associated with overall survival. **Conclusions**: Several nutritional and inflammatory markers improved from preoperative assessment to follow-up. Preoperative nutritional and inflammatory markers were associated with treatment discontinuation, particularly in the doublet therapy group. CT-derived muscle quality markers were associated with overall survival, suggesting prognostic relevance of muscle quality in this setting.

## Simple Summary

Patients with colorectal cancer may experience changes in nutritional status, inflammation, and body composition throughout the perioperative period and during subsequent adjuvant chemotherapy, but these changes are not well described. In this single-center retrospective study of 142 patients with Stage II/III colorectal cancer, several nutritional markers improved and inflammatory markers decreased between the preoperative assessment and 6 months after adjuvant chemotherapy initiation. Among patients receiving oxaliplatin-based doublet therapy, poorer preoperative nutritional and inflammatory status was associated with treatment discontinuation. In the monotherapy group, older age was the only factor significantly associated with discontinuation. In addition, lower preoperative muscle quality measured by computed tomography was associated with poorer overall survival, whereas muscle quantity was not. These findings suggest that nutritional, inflammatory, and muscle quality assessments may help identify patients who require closer supportive care.

## 1. Introduction

Colorectal cancer (CRC) is the third most common malignant tumor worldwide [1]. Following histological diagnosis and appropriate staging, treatment planning for nonmetastatic CRC is generally based on a multidisciplinary approach integrating surgical and oncological management with supportive care, including nutritional assessment [1,2,3,4]. The standard of care for patients with Stage III and high-risk Stage II CRC involves curative surgery followed by adjuvant chemotherapy to prevent recurrence and improve prognosis [2,3]. In Stage III CRC, oxaliplatin-based doublet therapy (CAPOX/FOLFOX) has demonstrated superiority over fluoropyrimidine monotherapy [2,3]. However, evidence supporting its superiority remains limited in older patients and those with high-risk Stage II disease, and treatment regimens should therefore be determined on a case-by-case basis.

Malnutrition is common in patients with cancer and may result from both cancer-related factors and anticancer treatments, highlighting the importance of early identification [4]. It is associated with a poor prognosis and an increased risk of postoperative complications [4,5,6]. Although nutritional assessment is recommended for all patients with cancer [4], no single tool has been established as the standard in CRC care [7,8]. Several nutritional and inflammatory markers are associated with short- and long-term outcomes in patients with CRC [7,9,10,11,12]. Body composition measures, including body mass index (BMI), muscle quantity, muscle quality, and adipose tissue distribution, are also clinically relevant, although associations with clinical outcomes have varied across cohorts [13,14,15,16,17,18,19,20]. In particular, methods for assessing muscle quality vary considerably across studies [20,21].

Despite these insights, longitudinal changes in nutritional, inflammatory, and body composition markers during adjuvant chemotherapy and their clinical significance remain poorly described in CRC [11,17]. Because adjuvant chemotherapy is delivered during recovery from surgery and may itself cause anorexia, gastrointestinal toxicity, and muscle loss [4,22], these markers may change throughout the treatment course, and a single preoperative assessment may not adequately capture their dynamic changes. Moreover, recent evidence suggests that computed tomography (CT)-derived body composition, particularly skeletal muscle radiodensity, may be associated with the completion of adjuvant chemotherapy in patients with CRC [23]. We therefore evaluated these changes and explored preoperative factors associated with treatment discontinuation and prognosis.

## 2. Materials and Methods

### 2.1. Study Design

**Figure 1:**
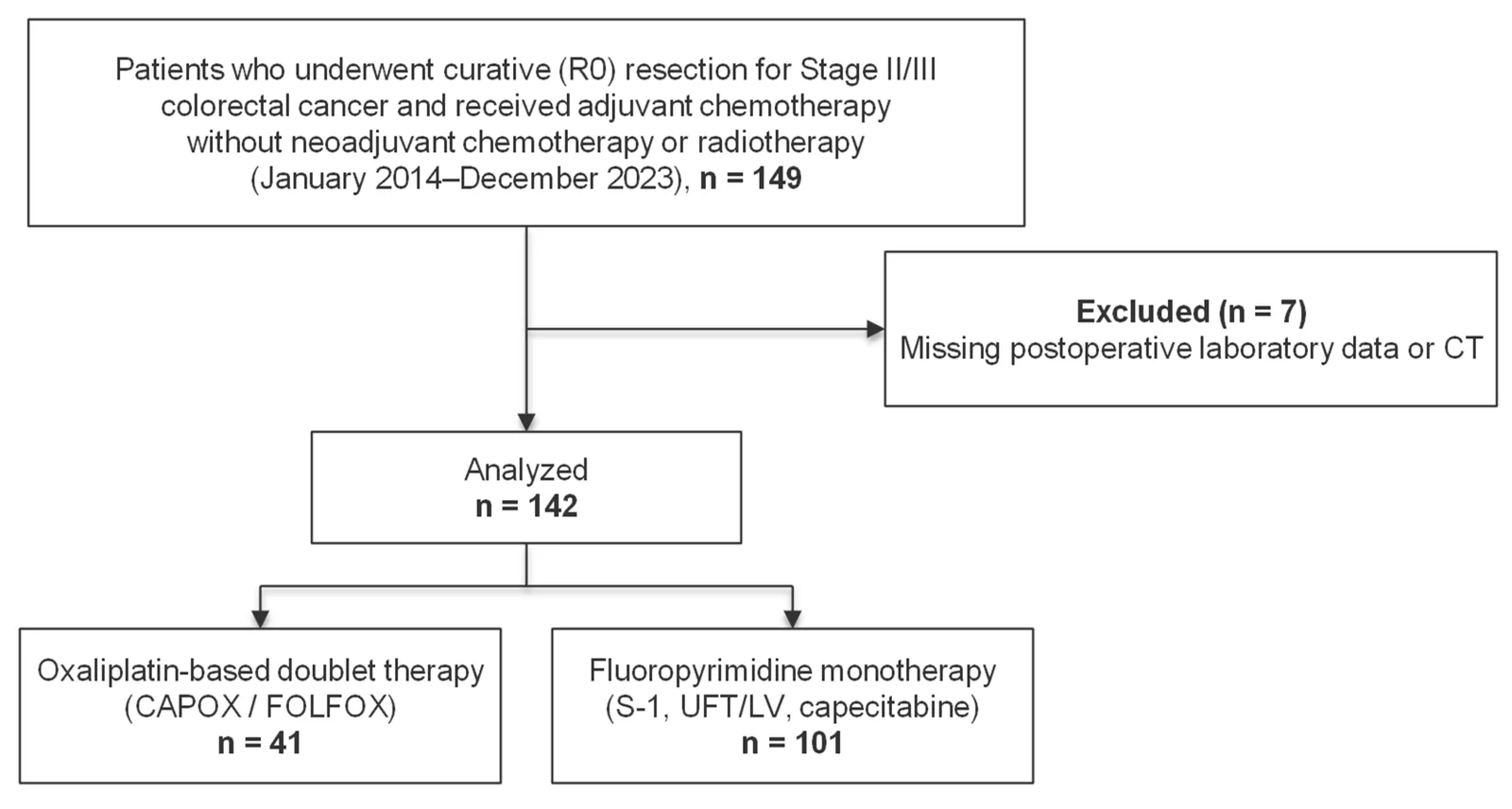
Flow diagram of patient selection.

In this single-center retrospective study, we analyzed patients who underwent curative surgery for Stage II/III CRC and received adjuvant chemotherapy at Fukushima Medical University Hospital from January 2014 to December 2023. The inclusion criteria were (i) histopathologically confirmed diagnosis of colorectal adenocarcinoma, (ii) curative surgery, and (iii) receipt of postoperative adjuvant chemotherapy. The exclusion criteria for this study were (i) non-curative surgery (microscopically or macroscopically positive margin), (ii) receipt of neoadjuvant chemotherapy and/or radiotherapy, and (iii) insufficient laboratory or CT data (Figure 1). This study was approved by the Institutional Ethics Committee of Fukushima Medical University (approval number 30148) for a retrospective analysis of the collected data in accordance with the ethical standards of the Declaration of Helsinki of the World Medical Association.

### 2.2. Nutritional Assessment, Counseling, and Rehabilitation

All patients underwent nutritional screening using the Malnutrition Universal Screening Tool (MUST) at hospital admission. Patients identified as being at nutritional risk were subsequently assessed for malnutrition. All patients received at least 30 min of nutritional counseling during their postoperative hospital stay before discharge. Post-discharge counseling was not routinely scheduled but was offered to patients considered at high risk of frailty or malnutrition. 

Perioperative rehabilitation was provided during the surgical hospitalization and was individualized according to each patient’s postoperative condition and pain. Rehabilitation included training in activities of daily living and exercise programs combining aerobic and resistance exercises, as tolerated. However, no standardized or structured rehabilitation program was routinely implemented after discharge. 

### 2.3. Adjuvant Chemotherapy

At our institution, postoperative adjuvant chemotherapy is offered after curative surgery to patients with pathological Stage III or high-risk Stage II CRC. General eligibility for treatment requires an Eastern Cooperative Oncology Group performance status of 0–1, adequate recovery from postoperative complications, preserved major organ function, and the absence of serious active comorbidities. 

The chemotherapy regimen is selected based on pathological Stage, patient age, and an overall risk assessment. For patients under 70 years of age with Stage III CRC, our standard approach is oxaliplatin-based doublet therapy with CAPOX (capecitabine and oxaliplatin) or FOLFOX (folinic acid plus 5-fluorouracil plus oxaliplatin). For patients aged 70 years or older and those with high-risk Stage II CRC, oral fluoropyrimidine monotherapy is preferred. Options include S-1 (tegafur, gimeracil, and oteracil potassium), UFT/LV (tegafur–uracil plus leucovorin), and capecitabine.

High-risk features for Stage II CRC were defined by established guidelines and included T4 tumor (serosal invasion or direct invasion of adjacent organs), tumor perforation, poorly differentiated tumor histology, lymphovascular invasion or perineural invasion, an inadequate lymph node yield (<12 lymph nodes sampled), and tumor budding. However, deficient mismatch repair (dMMR) and/or microsatellite instability-high (MSI-H) status is associated with a more favorable prognosis and confers little to no benefit from oral fluoropyrimidine monotherapy, which can be harmful [3,24,25,26]. Accordingly, we typically administer oxaliplatin-based doublet chemotherapy for Stage III CRC, regardless of dMMR/MSI-H status. For high-risk Stage II CRC, adjuvant therapy is generally not administered in patients with dMMR/MSI-H status. Final treatment decisions are made through shared decision-making.

### 2.4. Follow-Up Observation

During the adjuvant chemotherapy period, laboratory data were collected prior to each treatment cycle. Following curative surgery, all patients were enrolled in a 5-year surveillance program in accordance with the Japanese Society for Cancer of the Colon and Rectum (JSCCR) guidelines [2]. This protocol included measurement of serum tumor markers, including carcinoembryonic antigen (CEA) and carbohydrate antigen 19-9 (CA19-9), every 3 months. CT scans of the chest and abdomen were performed every 6 months, and surveillance colonoscopy was performed annually.

### 2.5. Data Collection

In this study, longitudinal assessment was defined as within-patient evaluation at two predefined time points: before curative surgery and approximately 6 months after initiation of adjuvant chemotherapy. The preoperative assessment was based on the most recent measurements obtained within 3 months before surgery, whereas the follow-up assessment used data from the first CT scan performed 6 months after initiation of adjuvant chemotherapy. Clinicopathological, molecular, and laboratory data, as well as information on adjuvant chemotherapy discontinuation and prognosis, were retrospectively obtained from the medical records. The Prognostic Nutritional Index (PNI), Geriatric Nutritional Risk Index (GNRI), hemoglobin–albumin–lymphocyte–platelet (HALP) score, neutrophil-to-lymphocyte ratio (NLR), platelet-to-lymphocyte ratio (PLR), psoas muscle index (PMI), psoas muscle density (PMD), and modified intramuscular adipose tissue content (mIMAC) were calculated according to previously reported methods [13,16,27,28]. 

### 2.6. CT-Derived Body Composition Analysis

**Figure 2:**
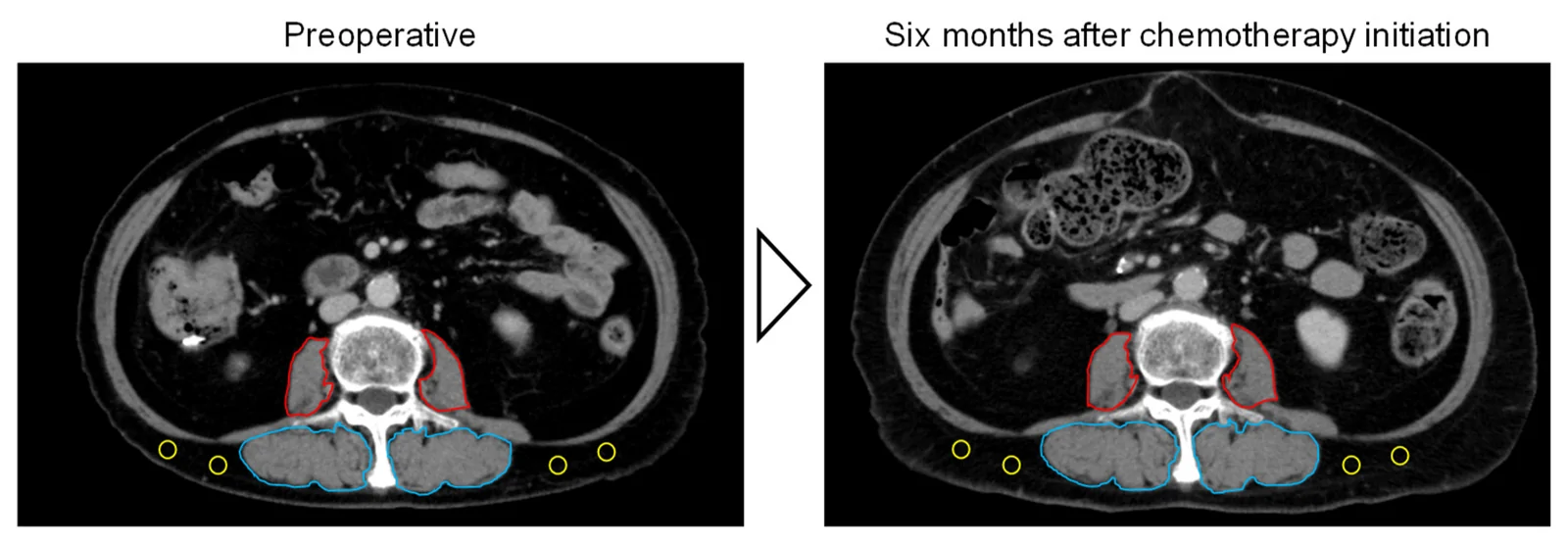
Representative axial CT images at the third lumbar vertebral level obtained preoperatively (**left**) and 6 months after initiation of adjuvant chemotherapy (**right**), illustrating the measurement of body composition parameters. The psoas muscle (red), multifidus muscle (blue), and subcutaneous fat (yellow) are segmented for analysis. The psoas muscle index (PMI) is calculated by dividing the total psoas muscle area (cm^2^) by the patient’s height squared (m^2^). The psoas muscle density (PMD) is measured as the mean attenuation of the muscle in Hounsfield units (HU) and reflects muscle quality. The modified Intramuscular Adipose Tissue Content (mIMAC) is defined as the mean CT value of the multifidus muscle minus that of the subcutaneous fat, serving as another key indicator of myosteatosis. The images are from a 74-year-old woman with pathological Stage III CRC who underwent curative resection and received adjuvant UFT/LV. She completed 6 courses of treatment. Body weight remained stable, from 63.0 kg before surgery to 63.4 kg at follow-up, with only minimal changes in the CT-derived body composition parameters (PMI, 4.50 to 4.28 cm^2^/m^2^; PMD, 46.5 to 47.7 HU; mIMAC, 147.5 to 146.3).

All CT images were reviewed by one of the authors (M.H.) using EV Insite software (version 3.17.0.8; PSP Corporation, Tokyo, Japan). The reviewer was blinded to patient outcomes and performed the radiologic measurements as follows. Freehand regions of interest (ROIs) were drawn around both psoas muscles and the multifidus muscle on the same CT slice, carefully excluding areas of obvious macroscopic fat infiltration. Additionally, CT attenuation values (Hounsfield units, HU) of subcutaneous fat were measured by placing four circular ROIs within homogeneous areas of subcutaneous fat at the same level, avoiding major vessels (Figure 2).

### 2.7. Statistical Analysis

Continuous variables were presented as the median and interquartile range (IQR), while categorical variables were presented as numbers and percentages (*n*, %). Changes in continuous variables between the preoperative and follow-up assessments were evaluated using the Wilcoxon signed-rank test for paired data. Within-patient changes were summarized using the Hodges–Lehmann (HL) estimator with 95% confidence intervals (CI). Comparisons of continuous variables between independent groups were performed using the Mann–Whitney U test. Analyses were performed for the overall cohort and separately for the doublet therapy and the monotherapy groups.

In the survival analyses, we assessed overall survival (OS) and disease-free survival (DFS). OS was defined as the time from curative surgery to death from any cause. DFS was defined as the time from curative surgery to disease recurrence or death from any cause, whichever occurred first. Patients without an event were censored at the earlier of the date of last follow-up and 5 years after resection. Kaplan–Meier curves were generated, and between-group differences were assessed using the log-rank test. Continuous variables were dichotomized at their median values. Sex-specific median values were used as cutoffs for CT-derived body composition markers (PMI, PMD, and mIMAC). 

All statistical analyses were performed using R software (version 4.5.1). The significance threshold was set at *p* < 0.05.

## 3. Results

### 3.1. Patient Characteristics

A total of 142 patients were included in this study. The median age was 67 years (interquartile range [IQR], 61–73 years), and 79 patients (55.6%) were male. Thirty-nine patients (27.5%) had high-risk Stage II disease, and 103 (72.5%) had Stage III disease. One hundred one patients (71.1%) received monotherapy, and 41 patients (28.9%) received doublet therapy. Dose reduction occurred in 31 patients (21.8%). Adjuvant chemotherapy was discontinued in 45 patients (31.7%). The reasons for discontinuation were adverse events in 36 patients (80.0%), cancer recurrence in 5 (11.1%), worsening comorbidities in 2 (4.4%), and patient decision in 2 (4.4%) (Table 1).

### 3.2. Longitudinal Change in Nutrition, Inflammation, and Body Composition

**Figure 3:**
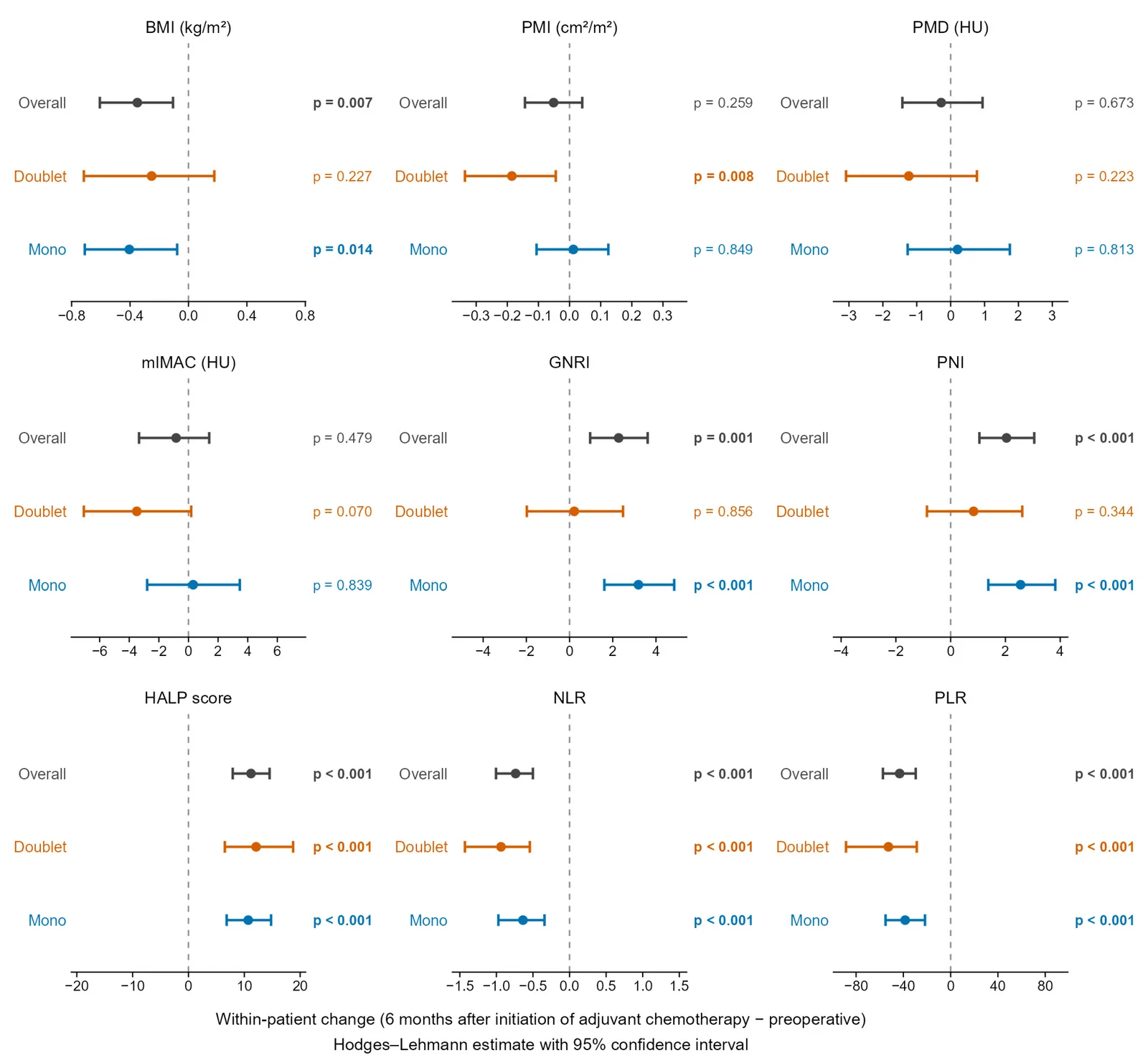
Within-patient changes in nutritional, inflammatory, and CT-derived body composition markers from preoperative assessment to approximately 6 months after initiation of adjuvant chemotherapy. Points represent Hodges–Lehmann estimates of the within-patient change (follow-up minus preoperative), and horizontal lines indicate 95% confidence intervals. Estimates are shown for the overall cohort, oxaliplatin-based doublet therapy group, and fluoropyrimidine monotherapy group. The vertical dashed line indicates no change. *p*-values were calculated using the Wilcoxon signed-rank test.

Nutritional and inflammatory markers were compared between the preoperative and follow-up assessments. In the overall cohort, GNRI (HL estimate of the within-patient change, +2.3; 95% CI, +1.0 to +3.6, *p* = 0.001), PNI (+2.0; +1.1 to +3.1, *p* < 0.001), and HALP score (+11.2; +7.9 to +14.5, *p* < 0.001) increased significantly. Conversely, BMI (−0.35 kg/m^2^; −0.61 to −0.11, *p* = 0.007), NLR (−0.74; −1.01 to −0.50, *p* < 0.001), and PLR (−43.2; −57.3 to −29.6, *p* < 0.001) decreased significantly. No significant changes were found in PMI, PMD, or mIMAC (Figure 3, Table S1).

In the doublet therapy group, HALP score increased significantly (+12.1; +6.5 to +18.7, *p* < 0.001), while significant decreases were observed in PMI (−0.19 cm^2^/m^2^; −0.34 to −0.04, *p* = 0.008), NLR (−0.94; −1.43 to −0.54, *p* < 0.001), and PLR (−52.6; −88.5 to −28.7, *p* < 0.001). No significant changes were observed in BMI, GNRI, PNI, PMD, or mIMAC (Table S2).

In the monotherapy group, significant increases were observed in GNRI (+3.2; +1.6 to +4.8, *p* < 0.001), PNI (+2.6; +1.4 to +3.8, *p* < 0.001), and the HALP score (+10.7; +6.8 to +14.8, *p* < 0.001). Conversely, BMI (−0.41 kg/m^2^; −0.71 to −0.08, *p* = 0.014), NLR (−0.64; −0.97 to −0.34, *p* < 0.001), and PLR (−38.5; −55.1 to −21.7, *p* < 0.001) decreased significantly (Table S3).

### 3.3. Factors Associated with Discontinuation

In the overall cohort, no factors were significantly associated with discontinuation of adjuvant chemotherapy. In the doublet therapy group (*n* = 41), patients who discontinued treatment (*n* = 12) had significantly lower preoperative GNRI (median, 102.4 vs. 107.1, *p* = 0.019), PNI (median, 46.6 vs. 50.3, *p* = 0.014), and HALP score (median, 18.8 vs. 34.5, *p* = 0.011), as well as significantly higher preoperative NLR (3.5 vs. 2.2, *p* = 0.045) and PLR (252.8 vs. 154.9, *p* = 0.020), than those who did not discontinue treatment (*n* = 29). In the monotherapy group (*n* = 101), patients who discontinued treatment (*n* = 33) were significantly older than those who did not (*n* = 68) (72.0 vs. 68.0 years, *p* = 0.018) (Table 2).

### 3.4. Survival Analyses

**Figure 4:**
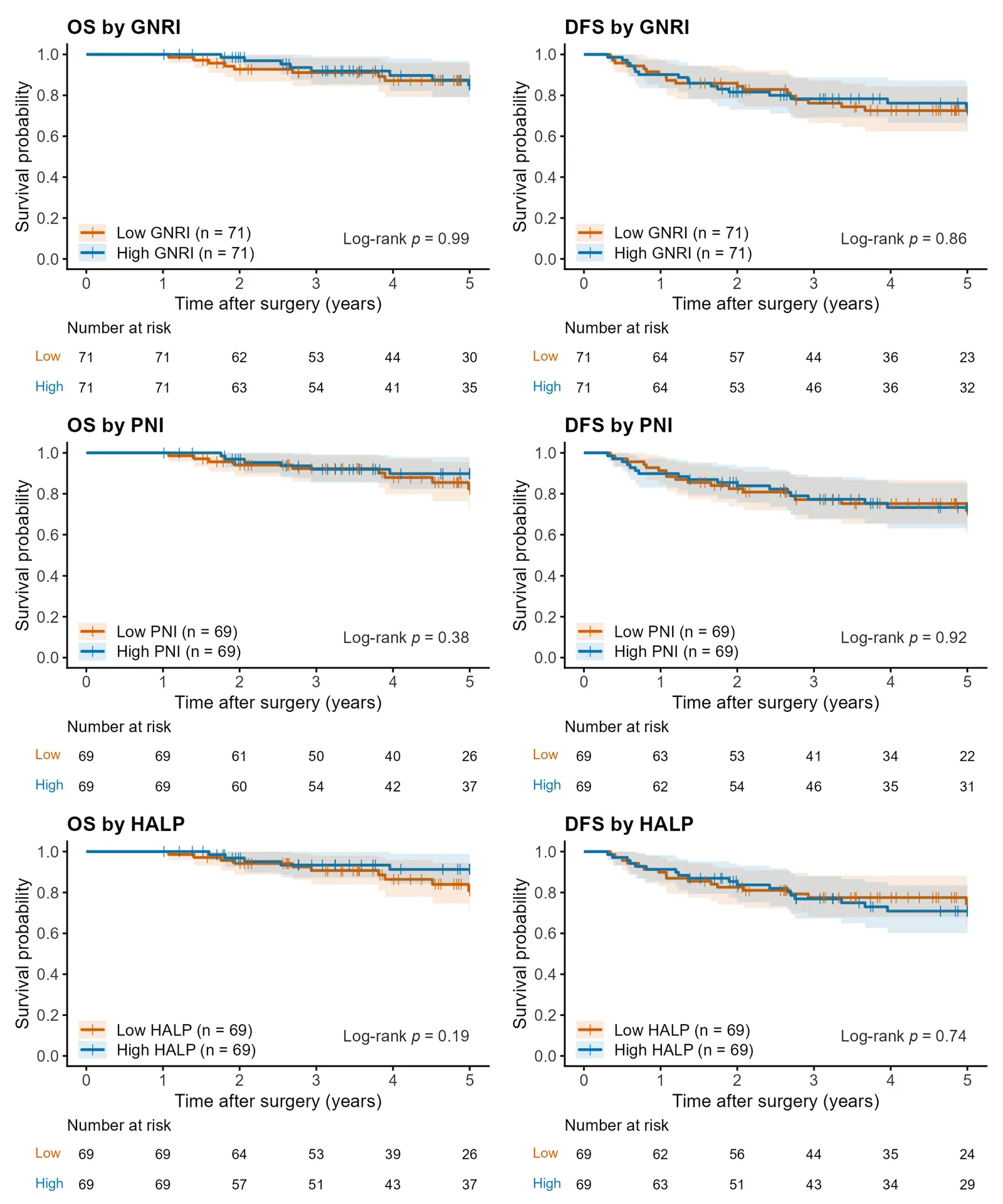
Kaplan–Meier analysis of overall survival (OS) and disease-free survival (DFS) stratified by preoperative nutritional markers. The log-rank test was used to compare survival curves. None of the nutritional markers (Geriatric Nutritional Risk Index [GNRI], Prognostic Nutritional Index [PNI], or hemoglobin–albumin–lymphocyte–platelet [HALP] score) were significantly associated with OS or DFS.

**Figure 5:**
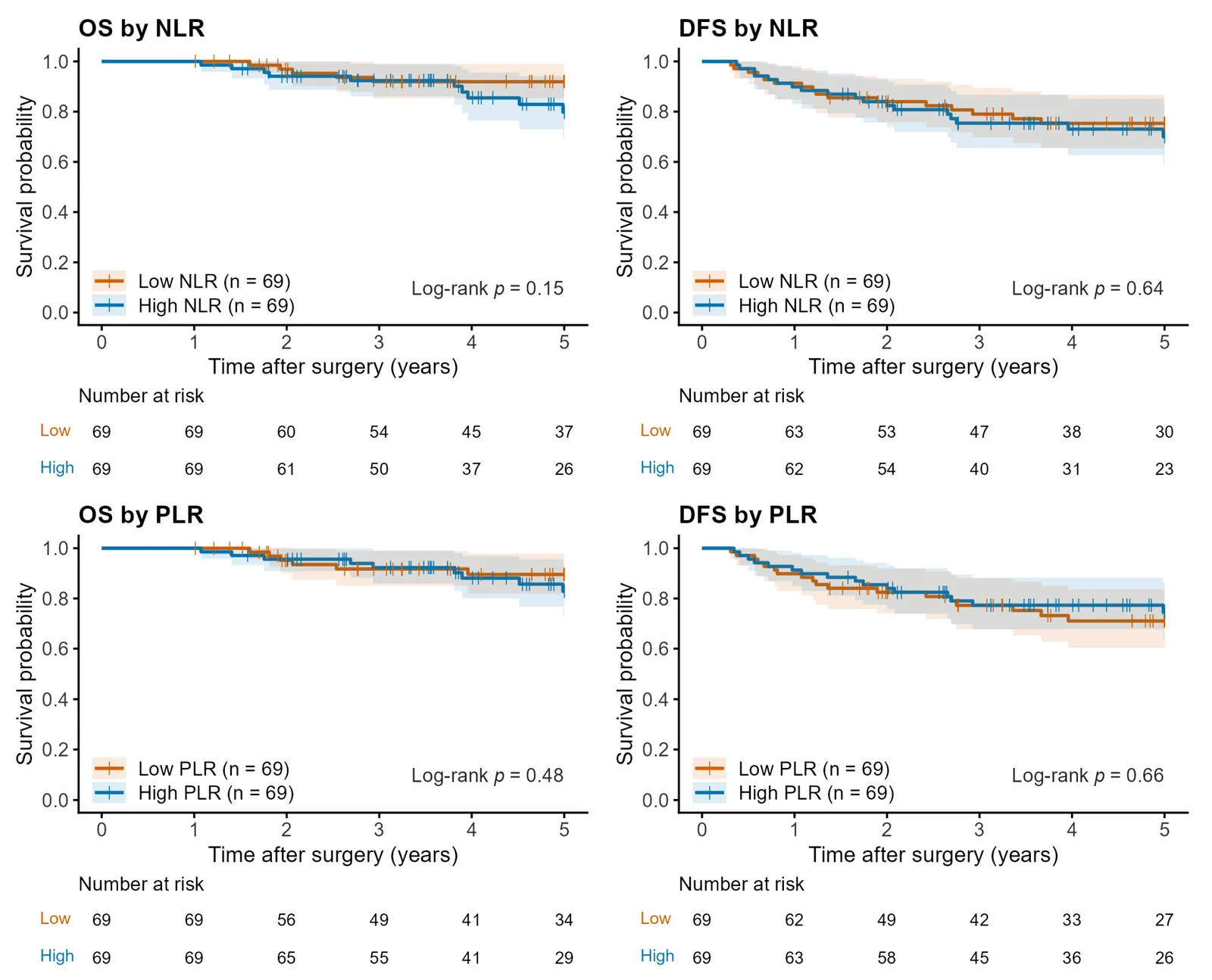
Kaplan–Meier analysis of overall survival (OS) and disease-free survival (DFS) stratified by preoperative inflammatory markers. The log-rank test was used to compare survival curves. None of the inflammatory markers (neutrophil-to-lymphocyte ratio [NLR] and platelet-to-lymphocyte ratio [PLR]) were significantly associated with OS or DFS.

**Figure 6:**
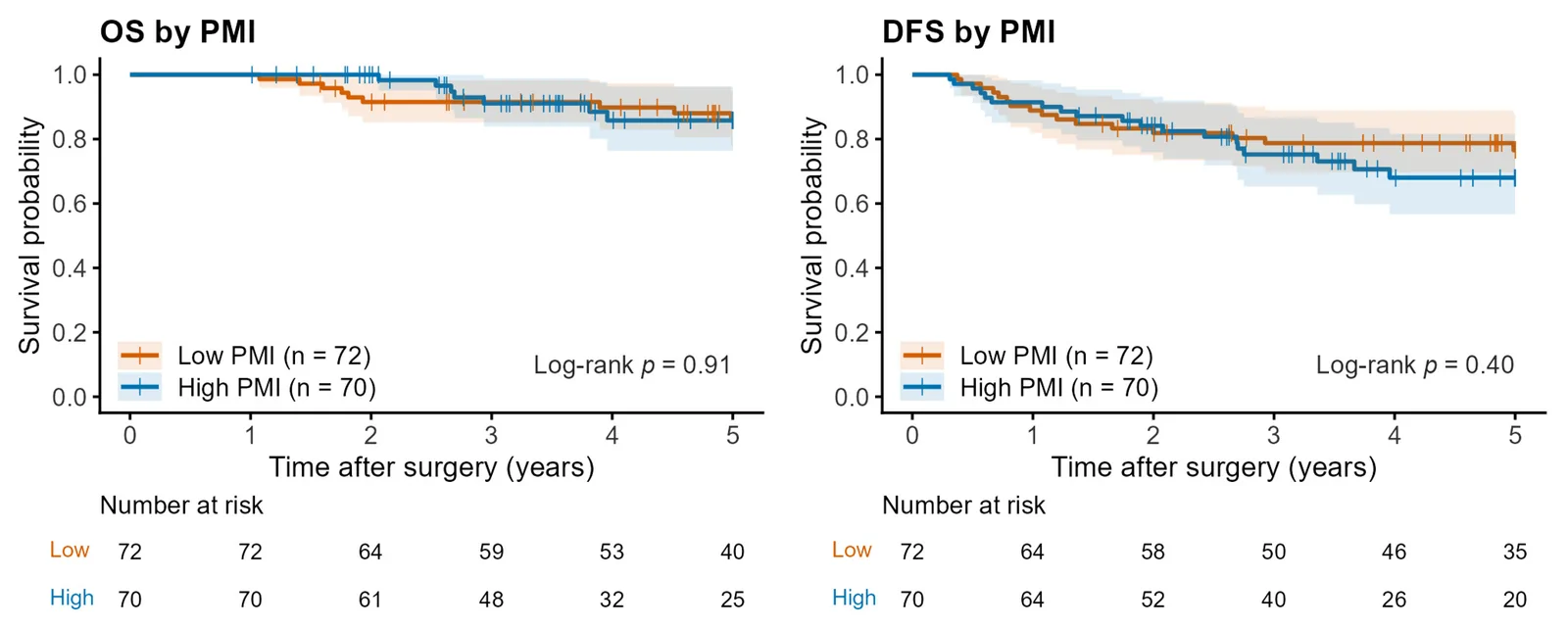

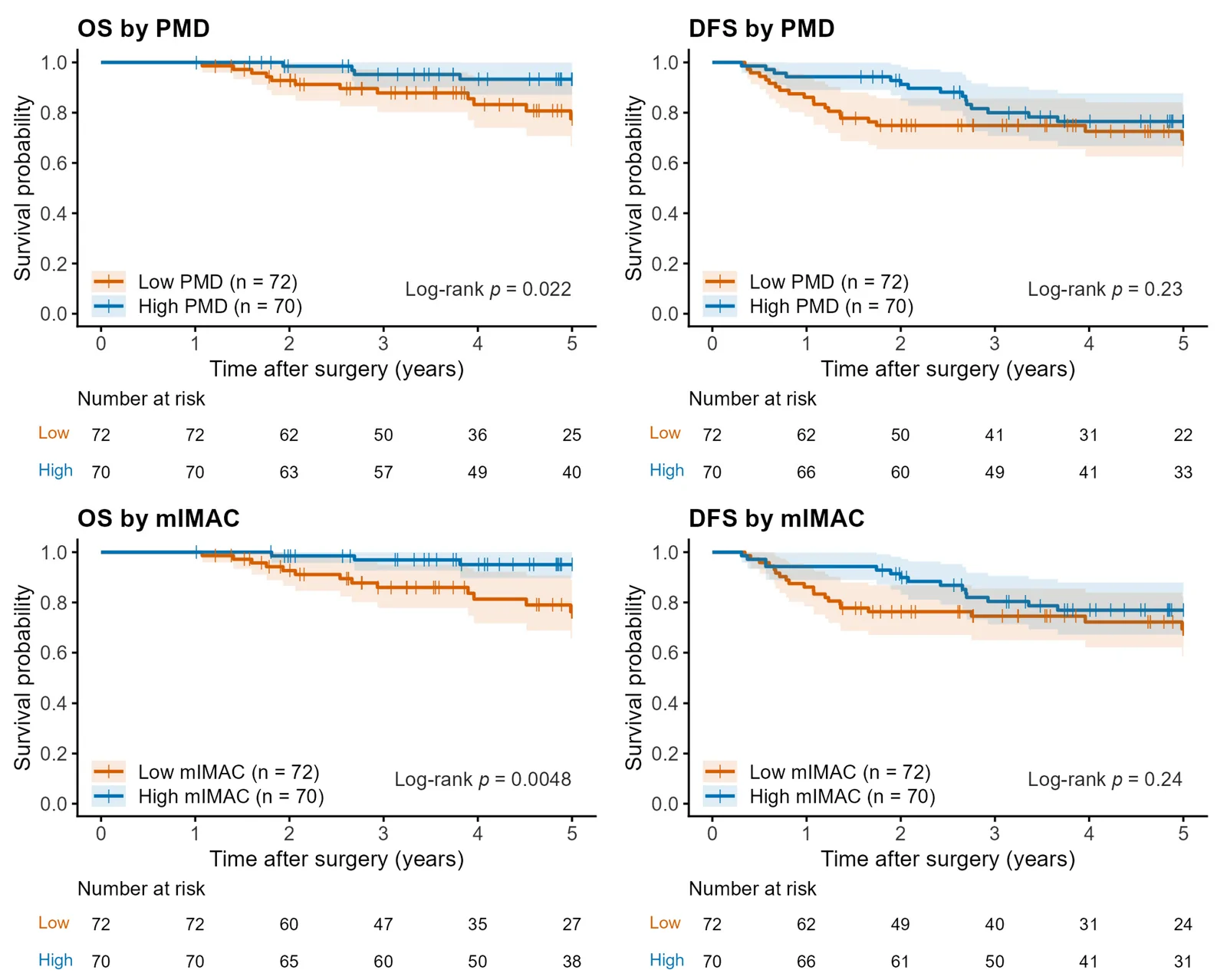
Kaplan–Meier analysis of overall survival (OS) and disease-free survival (DFS) stratified by preoperative body composition markers. The log-rank test was used to compare survival curves. Lower preoperative psoas muscle density (PMD) (*p* = 0.022) and lower modified intramuscular adipose tissue content (mIMAC) (*p* = 0.0048) were significantly associated with poorer OS. In contrast, none of the body composition markers were significantly associated with DFS.

Regarding DFS, none of the preoperative nutritional, inflammatory, or body composition markers were significantly associated with DFS (Figure 4, Figure 5 and Figure 6). For OS, lower preoperative PMD (*p* = 0.022) and lower mIMAC (*p* = 0.0048) were significantly associated with poorer OS (Figure 6). However, no significant association with OS was observed for the other markers (Figure 4, Figure 5 and Figure 6). In addition, discontinuation of adjuvant chemotherapy was not significantly associated with either DFS or OS (Figure S1).

## 4. Discussion

This study identified three main findings. First, several nutritional markers increased and inflammatory markers decreased between the preoperative and follow-up assessments, although PMI decreased in patients receiving doublet therapy. Second, preoperative nutritional and inflammatory status was associated with treatment discontinuation, particularly in the doublet therapy group, whereas older age was associated with discontinuation in the monotherapy group. Third, lower preoperative PMD and mIMAC were associated with poorer OS, whereas PMI was not, suggesting that muscle quality may be more prognostically relevant than muscle quantity in this setting. To our knowledge, this is one of the first studies to longitudinally assess nutritional, inflammatory, and CT-derived body composition markers in patients with CRC receiving adjuvant chemotherapy following curative surgery.

Malnutrition is common in patients with cancer and may result from both the disease itself and anticancer treatment [4]. In contrast, several nutritional and inflammatory markers improved between the preoperative and follow-up assessments in this study. There are two possible explanations. First, tumor resection may reduce disease-driven catabolism and systemic inflammation. Tumor-associated systemic inflammation and cachexia are driven in part by proinflammatory cytokines such as IL-6, IL-1β, and TNF-α, which suppress appetite through hypothalamic signaling and promote skeletal muscle catabolism; intratumoral detection of IL-6 and IL-1β has been correlated with cachexia [29]. Removal of the primary tumor may therefore attenuate tumor-associated inflammatory and catabolic processes. Consistent with this, patients with CRC show reduced postprandial muscle protein synthesis and an increased potential for muscle protein breakdown, both of which may be reversed by curative resection [30]. Postoperative recovery may therefore have contributed to the observed increases in GNRI, PNI, and HALP score and decreases in NLR and PLR. Nutritional counseling may also have played a role, because all patients received counseling before discharge and selected high-risk patients received additional counseling after discharge. However, nutritional intake and post-discharge interventions were not standardized or quantified, precluding assessment of their independent effects. A previous study also reported on the effects of nutritional counseling and intervention in patients with CRC after surgery [31].

In the doublet therapy group, PMI decreased significantly despite no significant change in BMI, suggesting that psoas muscle loss may occur without a corresponding change in BMI. In contrast, BMI decreased significantly in the overall cohort and the monotherapy group, whereas PMI remained stable. These findings suggest that changes in body weight or BMI do not necessarily parallel changes in skeletal muscle mass, as body weight and BMI do not distinguish skeletal muscle mass from adipose tissue and may also be influenced by fluid retention or edema [29]. Previous studies have reported weight gain after CRC diagnosis [32,33] or a high prevalence of excess body weight among CRC survivors [34]. However, weight change may differ according to the timing of assessment and pre-diagnosis weight loss [33]. The decrease in PMI observed in the doublet therapy group may be partly related to inflammation, as a prospective study reported increases in IL-8, TNF-α, and high-sensitivity C-reactive protein in patients who developed sarcopenia during chemotherapy [22]. Beyond these potential mechanisms, such muscle loss may also have clinical relevance because skeletal muscle loss during chemotherapy has been associated with severe treatment-related toxicity and poorer survival in older patients with metastatic CRC [35]. Taken together, these findings suggest the importance of closer monitoring of muscle mass in addition to body weight or BMI and early, targeted nutritional interventions in patients receiving oxaliplatin-based doublet therapy.

In contrast to prior reports, older age was not significantly associated with adjuvant chemotherapy discontinuation in the doublet therapy group [36,37]. This finding may partly reflect confounding by indication, because older patients were more likely to receive monotherapy. This tendency is well documented in population-based studies, which indicate that older adults are less likely to receive doublet therapy and may receive shorter treatment durations [36]. In the doublet therapy group, several preoperative nutritional and inflammatory markers, rather than age, were associated with treatment discontinuation. Although PNI and GNRI have previously been studied in relation to treatment tolerability [11,17], HALP score, NLR, and PLR were also associated with discontinuation in our cohort. With respect to CT-derived body composition, Yokoi et al. recently reported that low skeletal muscle radiodensity, a marker of myosteatosis, was independently associated with failure to complete adjuvant CAPOX therapy in patients with CRC [23]. Conversely, preoperative PMD and mIMAC were not associated with treatment discontinuation in our cohort. This discrepancy may reflect differences in the muscle compartments assessed (total skeletal muscle at the L3 level versus the psoas and multifidus muscles), differences in how muscle radiodensity was analyzed (lowest-quartile categorization versus analysis as a continuous variable), and the limited sample size of our doublet therapy subgroup. In contrast, older age was the only factor associated with discontinuation in the monotherapy group. This finding should be interpreted with caution. Although a pooled analysis of seven randomized trials showed that older patients derive a similar benefit from fluorouracil-based adjuvant therapy to that observed in younger patients [38], factors associated with treatment continuation in older patients remain less well described.

In our survival analyses, the preoperative muscle quality markers PMD and mIMAC were both significantly associated with OS. These CT-derived markers reflect myosteatosis, with lower PMD and mIMAC indicating greater myosteatosis and poorer muscle quality. Consistent with prior reports, mIMAC has been associated with both short- and long-term outcomes in patients with gastrointestinal cancers [13,39]. PMD is a CT-based marker of muscle quality that reflects replacement of contractile fibers by adipose and connective tissue and correlates with physical performance [40,41,42]. In colorectal and other gastrointestinal cancers, low PMD has been associated with higher postoperative complication rates and poorer long-term outcomes, including worse overall survival [40,41,42]. In contrast, PMI was not significantly associated with OS in our cohort. Similarly, a recent study of patients with upper gastrointestinal tract cancer suggested that impaired muscle quality, as reflected by low skeletal muscle density, may have greater prognostic relevance for OS than reduced muscle quantity alone [43]. These findings suggest that muscle quality may provide more clinically relevant prognostic information than muscle quantity in this setting.

This study did not assess physical function or activity, and no ongoing structured rehabilitation program was implemented after discharge. Therefore, the contribution of these factors to prognosis remains unclear. A previous study showed that physical activity levels during chemotherapy cycles were low, particularly in the early phase of each cycle [44]. There is also evidence that exercise-based rehabilitation during adjuvant chemotherapy is feasible and safe [45], and that home-based exercise after completion of adjuvant treatment can improve quality of life and psychological health in CRC survivors [46]. Notably, a recent phase 3 trial reported that structured exercise after adjuvant chemotherapy improved disease-free survival and was associated with longer OS in colon cancer [47], while a recent randomized clinical trial reported that multimodal prehabilitation before CRC surgery reduced severe postoperative complications and enhanced functional recovery [48]. Together, these findings support the potential clinical value of exercise-based interventions at different stages of CRC treatment, although whether such interventions preserve muscle quality remains to be determined. Future studies are therefore needed to determine whether targeted interventions, such as structured exercise, may be particularly beneficial for patients with poor preoperative muscle quality or impaired physical function.

This study has several limitations. First, this was a retrospective, single-center study, which may limit generalizability. Second, heterogeneity in disease Stage, regimen, and treatment duration may have introduced selection bias and confounding by indication. Third, the relatively small sample size precluded multivariable regression analysis, limiting our ability to account for potential confounders. Fourth, the effects of adjuvant chemotherapy could not be clearly separated from those of the preceding surgery, because gastrointestinal surgery and postoperative complications may adversely affect nutritional status through reduced oral intake, stress-related catabolism, and impaired gastrointestinal function [49]. Fifth, our CT-derived body composition analysis focused on muscle quantity and quality and did not assess visceral adiposity, which has been associated with postoperative complications after CRC surgery [21]. Finally, nutritional counseling, dietary intake, and physical activity were neither standardized nor quantified, and may therefore have influenced the observed changes.

Future prospective multicenter studies with larger sample sizes and appropriate adjustment for disease Stage and adjuvant treatment regimen are warranted. Serial assessments of nutritional, inflammatory, and CT-derived body composition markers should be performed before surgery, after surgery before adjuvant chemotherapy, and after chemotherapy, together with longitudinal evaluation of nutritional status, dietary intake, physical activity, and physical function. Such studies may help disentangle the effects of surgery from those of chemotherapy and identify modifiable factors associated with treatment completion and long-term outcomes.

## 5. Conclusions

To our knowledge, this study is among the first to longitudinally evaluate nutritional, inflammatory, and CT-derived body composition markers within the same cohort of patients with Stage II/III CRC undergoing curative surgery and subsequent adjuvant chemotherapy, and to assess their associations with treatment discontinuation according to chemotherapy regimen. During the treatment course, several nutritional markers improved and inflammatory markers decreased. However, PMI declined in patients receiving oxaliplatin-based doublet therapy despite relatively stable body weight, suggesting that loss of skeletal muscle mass may not be adequately reflected by changes in BMI alone. In patients receiving doublet therapy, lower preoperative GNRI, PNI, and HALP score, as well as higher NLR and PLR, were associated with treatment discontinuation. In those receiving monotherapy, only older age was associated with treatment discontinuation. With respect to long-term prognosis, lower preoperative PMD and mIMAC were associated with poorer OS, whereas PMI was not. These findings suggest that muscle quality may provide more clinically relevant prognostic information than muscle quantity in this setting. Assessing nutritional, inflammatory, and body composition markers may also help identify patients who require closer supportive care, such as continued nutritional counseling and longitudinal body composition monitoring. In particular, a preoperative assessment of these markers could help identify patients at higher risk for discontinuing treatment before oxaliplatin-based doublet therapy is initiated, enabling early consideration of supportive interventions. Further prospective studies are needed to determine whether targeted nutritional, exercise, and body composition monitoring strategies can improve treatment completion and long-term outcomes.

## Figures and Tables

**Table 1:** Patient characteristics and details of adjuvant chemotherapy including regimens and discontinuation.

Characteristics	All Patients, *n* = 142
Age (years old), median (IQR)	67 (61, 73)
Sex, *n* (%)	
Male	79 (55.6)
Female	63 (44.4)
Stage, *n* (%)	
II	39 (27.5)
III	103 (72.5)
Postoperative adjuvant therapy regimen, *n* (%)	
S-1 6 month	1 (0.7)
UFT/LV 6 month	72 (50.7)
Capecitabine 6 month	28 (19.7)
CAPOX 3 month	12 (8.5)
CAPOX 6 month	28 (19.7)
FOLFOX 3 month	0 (0.0)
FOLFOX 6 month	1 (0.7)
Dose reduction in adjuvant chemotherapy, *n* (%)	31 (21.8)
Adjuvant chemotherapy discontinuation, *n* (%)	45 (31.7)
Reasons for discontinuation, *n* (%)	
Adverse event	36 (80.0)
Cancer recurrence	5 (11.1)
Worsening comorbidity	2 (4.4)
Patient decision/voluntary withdrawal	2 (4.4)

S-1: tegafur/gimeracil/oteracil potassium; UFT/LV: tegafur/uracil plus leucovorin; CAPOX: capecitabine plus oxaliplatin; FOLFOX: leucovorin (folinic acid) plus 5-fluorouracil plus oxaliplatin; IQR: interquartile range.

**Table 2:** Preoperative characteristics according to discontinuation of adjuvant chemotherapy.

Characteristics	Doublet Therapy (*n* = 41)			Monotherapy (*n* = 101)		
	No Discontinuation (*n* = 29)	Discontinuation (*n* = 12)	*p*-Value	No Discontinuation (*n* = 68)	Discontinuation (*n* = 33)	*p*-Value
Age (years old)	64.0 [57.0–67.0]	56.5 [50.5–66.3]	0.329	68.0 [63.8–73.0]	72.0 [69.0–76.0]	0.018
BMI (kg/m^2^)	23.7 [20.8–25.6]	21.3 [20.1–24.0]	0.330	21.9 [19.9–23.9]	23.0 [20.7–25.5]	0.077
PMI (cm^2^/m^2^)	5.7 [4.5–6.7]	4.1 [3.2–5.7]	0.152	4.5 [3.7–5.4]	4.7 [3.6–5.6]	0.567
PMD (HU)	45.1 [38.8–50.2]	43.5 [37.7–49.1]	0.886	43.3 [33.6–49.9]	40.1 [35.8–43.6]	0.263
mIMAC	142.9 [132.1–149.2]	144.3 [136.9–152.5]	0.390	130.9 [118.0–142.7]	133.3 [120.6–141.3]	0.742
GNRI	107.1 [101.1–112.1]	102.4 [95.3–103.7]	0.019	101.5 [94.7–106.6]	101.5 [94.7–109.2]	0.476
PNI	50.3 [47.6–52.3]	46.6 [41.2–48.5]	0.014	47.3 [43.6–50.6]	46.8 [43.7–49.7]	0.631
HALP score	34.5 [23.1–52.1]	18.8 [13.9–27.5]	0.011	25.6 [17.2–38.9]	25.0 [17.5–39.8]	0.979
NLR	2.2 [1.8–3.0]	3.5 [2.6–4.0]	0.045	2.5 [1.9–3.8]	2.3 [1.7–3.6]	0.295
PLR	154.9 [117.6–206.9]	252.8 [179.9–309.2]	0.020	170.9 [131.7–246.3]	177.3 [126.8–230.0]	0.565

All nutritional, inflammatory, and body composition markers were assessed preoperatively. Values are medians [interquartile range]. *p*-values were calculated using the Mann–Whitney U test for comparisons between the discontinuation and no-discontinuation groups. Doublet therapy = CAPOX or FOLFOX; monotherapy = S-1, UFT/LV or capecitabine. BMI: body mass index; PMI: psoas muscle index; PMD: psoas muscle density; mIMAC: modified intramuscular adipose tissue content; GNRI: Geriatric Nutritional Risk Index; PNI: Prognostic Nutritional Index; HALP score: hemoglobin–albumin–lymphocyte–platelet score; NLR: neutrophil-to-lymphocyte ratio; PLR: platelet-to-lymphocyte ratio.

## Data Availability

The data underlying this study are available from the corresponding author on reasonable request, subject to institutional and ethical restrictions.

## Supplementary Materials

The following supporting information can be downloaded at: https://www.mdpi.com/article/10.3390/cancers18172886/s1, Figure S1. Kaplan-Meier analysis of overall survival (OS) and disease-free survival (DFS) stratified by adjuvant chemotherapy discontinuation. The log-rank test was used to compare survival curves. Discontinuation of adjuvant chemotherapy was not significantly associated with either DFS or OS; Table S1. Changes in body composition and nutritional/inflammatory indices between the preoperative and follow-up assessments 6 months after initiation of adjuvant chemotherapy in the overall cohort (n = 142); Table S2. Changes in body composition and nutritional/inflammatory indices between the preoperative and follow-up assessments 6 months after initiation of adjuvant chemotherapy in the doublet therapy group (n = 41); Table S3. Changes in body composition and nutritional/inflammatory indices between the preoperative and follow-up assessments 6 months after initiation of adjuvant chemotherapy in the monotherapy group (n = 101); Table S4. Preoperative characteristics according to adjuvant chemotherapy discontinuation.

## Abbreviations

The following abbreviations are used in this manuscript:

BMI	Body mass index
CA19-9	Carbohydrate antigen 19-9
CAPOX	Capecitabine plus oxaliplatin
CEA	Carcinoembryonic antigen
CI	Confidence interval
CRC	Colorectal cancer
CT	Computed tomography
DFS	Disease-free survival
dMMR	Deficient mismatch repair
FOLFOX	Folinic acid plus 5-fluorouracil plus oxaliplatin
GNRI	Geriatric Nutritional Risk Index
HALP	Hemoglobin–albumin–lymphocyte–platelet (score)
HL	Hodges–Lehmann (estimator)
HU	Hounsfield units
IQR	Interquartile range
JSCCR	Japanese Society for Cancer of the Colon and Rectum
mIMAC	Modified intramuscular adipose tissue content
MSI-H	Microsatellite instability-high
MUST	Malnutrition Universal Screening Tool
NLR	Neutrophil-to-lymphocyte ratio
OS	Overall survival
PLR	Platelet-to-lymphocyte ratio
PMD	Psoas muscle density
PMI	Psoas muscle index
PNI	Prognostic Nutritional Index
ROI	Region of interest
S-1	Tegafur, gimeracil, and oteracil potassium
UFT/LV	Tegafur–uracil plus leucovorin
