# Supplementary material for: Longitudinal Changes in Nutritional, Inflammatory, and CT-Derived Body Composition Markers During Adjuvant Chemotherapy for Stage II/III Colorectal Cancer
Source: Cancers (Basel). 2026 Sep 6;18(17):2886. doi: 10.3390/cancers18172886 (PMC13565724; doi:10.3390/cancers18172886)
Supplement: Supplementary file 1 [file cancers-18-02886-s001.zip › cancers-4525616-supplementary.pdf]

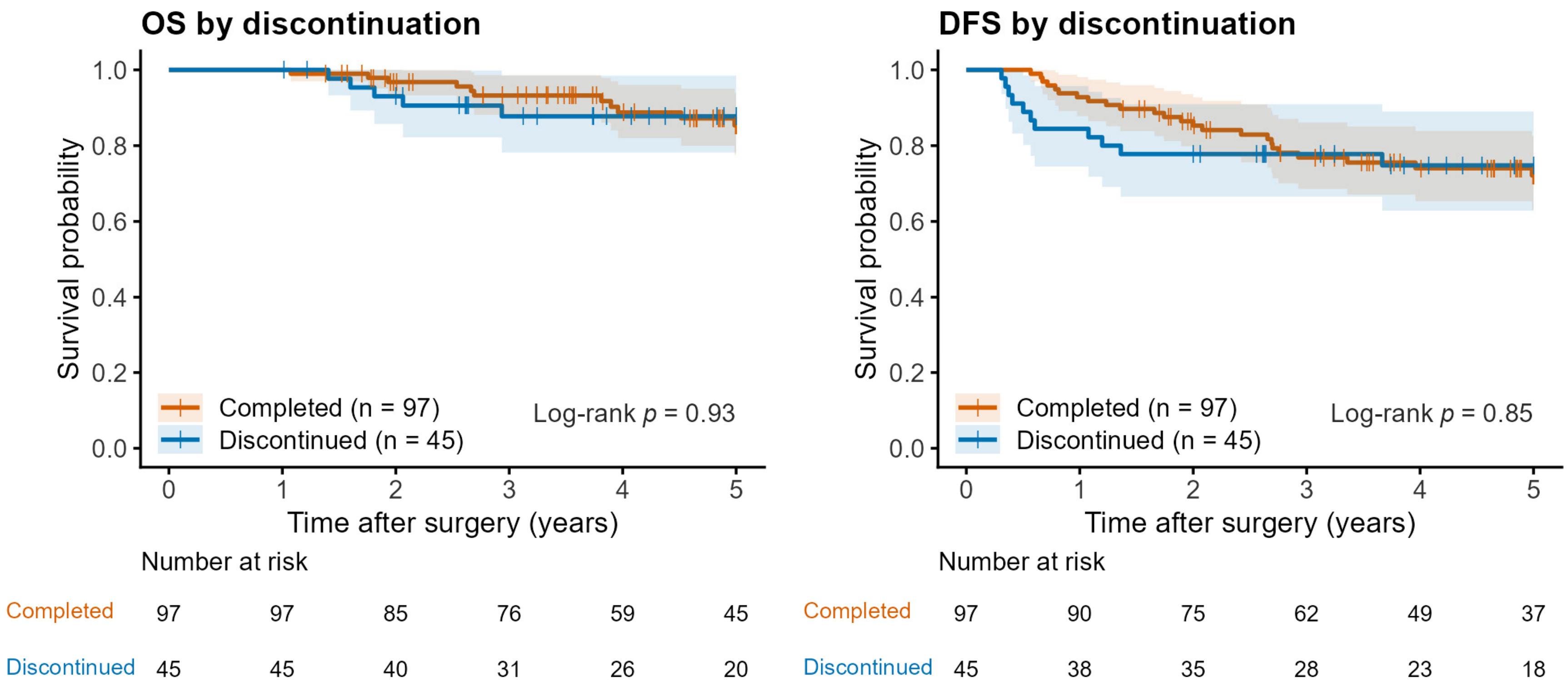

Figure S1. Kaplan-Meier analysis of overall survival (OS) and disease-free survival (DFS) stratified by adjuvant chemotherapy discontinuation. The log-rank test was used to compare survival curves. Discontinuation of adjuvant chemotherapy was not significantly associated with either DFS or OS.

Table S1. Changes in body composition and nutritional/inflammatory indices between the preoperative and follow-up assessments 6 months after initiation of adjuvant chemotherapy in the overall cohort (*n* = 142).

**Characteristics**	**Preoperative**	**Follow-up**	**HL estimate (95% CI)**	***p*-value**
BMI (kg/m²)	22.5 [20.2–24.8]	22.7 [20.0–24.3]	−0.35 (−0.61 to −0.11)	0.007
PMI (cm²/m²)	4.7 [3.6–5.7]	4.6 [3.5–5.6]	−0.05 (−0.14 to +0.04)	0.259
PMD (HU)	42.6 [36.3–48.5]	42.0 [36.3–48.0]	−0.3 (−1.4 to +0.9)	0.673
mIMAC	134.1 [122.0–145.2]	135.0 [125.9–144.5]	−0.8 (−3.3 to +1.4)	0.479
GNRI	103.3 [95.8–108.3]	104.1 [97.9–111.1]	+2.3 (+1.0 to +3.6)	0.001
PNI	47.6 [44.5–50.5]	49.1 [46.6–53.0]	+2.0 (+1.1 to +3.1)	<0.001
HALP score	26.1 [17.5–40.4]	38.7 [28.5–51.5]	+11.2 (+7.9 to +14.5)	<0.001
NLR	2.5 [1.8–3.7]	1.8 [1.3–2.6]	−0.74 (−1.01 to −0.50)	<0.001
PLR	177.3 [129.3–249.4]	137.3 [103.6–176.0]	−43.2 (−57.3 to −29.6)	<0.001

Values are medians [interquartile range]. HL estimates indicate Hodges–Lehmann estimates of the within-patient change (follow-up value minus preoperative value) with 95% confidence intervals (CI). *p*-values were calculated using the Wilcoxon signed-rank test for paired comparisons between preoperative and follow-up values. Monotherapy = S-1, UFT/LV or capecitabine; doublet therapy = CAPOX or FOLFOX. BMI: body mass index; PMI: psoas muscle index; PMD: psoas muscle density; mIMAC: modified intramuscular adipose tissue content; GNRI: Geriatric Nutritional Risk Index; PNI: Prognostic Nutritional Index; HALP score: hemoglobin–albumin–lymphocyte–platelet score; NLR: neutrophil-to-lymphocyte ratio; PLR: platelet-to-lymphocyte ratio.

Table S2. Changes in body composition and nutritional/inflammatory indices between the preoperative and follow-up assessments 6 months after initiation of adjuvant chemotherapy in the doublet therapy group (n = 41).

**Characteristics**	**Preoperative**	**Follow-up**	**HL estimate (95% CI)**	***p*-value**
BMI (kg/m^2)	23.3 [20.1–25.5]	22.9 [21.1–25.1]	−0.25 (−0.72 to +0.18)	0.227
PMI (cm^2/m^2)	5.4 [3.7–6.7]	4.6 [3.4–6.4]	−0.19 (−0.34 to −0.04)	0.008
PMD (HU)	45.1 [38.4–50.2]	44.7 [36.9–49.4]	−1.2 (−3.1 to +0.8)	0.223
mIMAC	143.4 [134.2–151.4]	139.7 [132.1–146.4]	−3.5 (−7.1 to +0.2)	0.070
GNRI	105.1 [99.0–111.5]	104.9 [96.1–110.9]	+0.2 (−2.0 to +2.5)	0.856
PNI	49.2 [45.6–51.4]	48.8 [47.2–52.8]	+0.8 (−0.9 to +2.6)	0.344
HALP score	28.7 [19.3–46.7]	41.0 [32.9–57.0]	+12.1 (+6.5 to +18.7)	<0.001
NLR	2.5 [1.8–3.6]	1.7 [1.4–2.1]	−0.94 (−1.43 to −0.54)	<0.001
PLR	178.7 [131.9–260.9]	121.3 [99.9–169.7]	−52.6 (−88.5 to −28.7)	<0.001

Values are medians [interquartile range]. HL estimates indicate Hodges–Lehmann estimates of the within-patient change (follow-up value minus preoperative value) with 95% confidence intervals (CI). *p*-values were calculated using the Wilcoxon signed-rank test for paired comparisons between preoperative and follow-up values. Monotherapy = S-1, UFT/LV or capecitabine; doublet therapy = CAPOX or FOLFOX. BMI: body mass index; PMI: psoas muscle index; PMD: psoas muscle density; mIMAC: modified intramuscular adipose tissue content; GNRI: Geriatric Nutritional Risk Index; PNI: Prognostic Nutritional Index; HALP score: hemoglobin–albumin–lymphocyte–platelet score; NLR: neutrophil-to-lymphocyte ratio; PLR: platelet-to-lymphocyte ratio.

Table S3. Changes in body composition and nutritional/inflammatory indices between the preoperative and follow-up assessments 6 months after initiation of adjuvant chemotherapy in the monotherapy group (n = 101).

**Characteristics**	**Preoperative**	**Follow-up**	**HL estimate (95% CI)**	***p*-value**
BMI (kg/m^2)	22.3 [20.2–24.5]	22.6 [20.0–24.2]	−0.41 (−0.71 to −0.08)	0.014
PMI (cm^2/m^2)	4.6 [3.6–5.5]	4.6 [3.5–5.4]	+0.01 (−0.11 to +0.12)	0.849
PMD (HU)	41.7 [34.3–48.4]	41.6 [35.9–47.8]	+0.2 (−1.3 to +1.7)	0.813
mIMAC	131.8 [118.9–142.7]	132.7 [120.0–143.7]	+0.3 (−2.8 to +3.5)	0.839
GNRI	101.5 [94.8–107.4]	103.8 [98.5–111.5]	+3.2 (+1.6 to +4.8)	<0.001
PNI	47.2 [43.9–49.9]	49.1 [46.3–53.2]	+2.6 (+1.4 to +3.8)	<0.001
HALP score	25.5 [17.2–39.4]	36.0 [28.4–49.3]	+10.7 (+6.8 to +14.8)	<0.001
NLR	2.4 [1.8–3.8]	2.0 [1.3–2.9]	−0.64 (−0.97 to −0.34)	<0.001
PLR	175.7 [128.6–249.3]	142.2 [109.2–180.0]	−38.5 (−55.1 to −21.7)	<0.001

Values are medians [interquartile range]. HL estimates indicate Hodges–Lehmann estimates of the within-patient change (follow-up value minus preoperative value) with 95% confidence intervals (CI). *p*-values were calculated using the Wilcoxon signed-rank test for paired comparisons between preoperative and follow-up values. Monotherapy = S-1, UFT/LV or capecitabine; doublet therapy = CAPOX or FOLFOX. BMI: body mass index; PMI: psoas muscle index; PMD: psoas muscle density; mIMAC: modified intramuscular adipose tissue content; GNRI: Geriatric Nutritional Risk Index; PNI: Prognostic Nutritional Index; HALP score: hemoglobin–albumin–lymphocyte–platelet score; NLR: neutrophil-to-lymphocyte ratio; PLR: platelet-to-lymphocyte ratio.

Table S4. Preoperative characteristics according to adjuvant chemotherapy discontinuation.

Characteristics	Overall (n = 142)			Doublet therapy (n = 41)			Monotherapy (n = 101)		
	No discontinuation (n = 97)	Discontinuation (n = 45)	p-Value	No discontinuation (n = 29)	Discontinuation (n = 12)	p-Value	No discontinuation (n = 68)	Discontinuation (n = 33)	p-Value
Age (years old)	66.0 [63.0–72.0]	70.0 [61.0–75.0]	0.090	64.0 [57.0–67.0]	56.5 [50.5–66.3]	0.329	68.0 [63.8–73.0]	72.0 [69.0–76.0]	0.018
BMI (kg/m²)	22.6 [19.9–24.3]	22.5 [20.6–25.0]	0.379	23.7 [20.8–25.6]	21.3 [20.1–24.0]	0.330	21.9 [19.9–23.9]	23.0 [20.7–25.5]	0.077
PMI (cm²/m²)	4.8 [3.7–5.8]	4.6 [3.6–5.6]	0.605	5.7 [4.5–6.7]	4.1 [3.2–5.7]	0.152	4.5 [3.7–5.4]	4.7 [3.6–5.6]	0.567
PMD (HU)	44.6 [34.3–50.2]	40.5 [36.9–45.4]	0.274	45.1 [38.8–50.2]	43.5 [37.7–49.1]	0.886	43.3 [33.6–49.9]	40.1 [35.8–43.6]	0.263
mIMAC	132.8 [121.2–145.1]	137.0 [122.4–145.2]	0.625	142.9 [132.1–149.2]	144.3 [136.9–152.5]	0.390	130.9 [118.0–142.7]	133.3 [120.6–141.3]	0.742
GNRI	103.9 [95.8–108.2]	102.3 [94.7–107.3]	0.579	107.1 [101.1–112.1]	102.4 [95.3–103.7]	0.019	101.5 [94.7–106.6]	101.5 [94.7–109.2]	0.476
PNI	48.2 [44.8–51.3]	46.8 [43.2–49.7]	0.070	50.3 [47.6–52.3]	46.6 [41.2–48.5]	0.014	47.3 [43.6–50.6]	46.8 [43.7–49.7]	0.631
HALP score	28.5 [18.8–40.8]	23.7 [14.5–36.1]	0.139	34.5 [23.1–52.1]	18.8 [13.9–27.5]	0.011	25.6 [17.2–38.9]	25.0 [17.5–39.8]	0.979
NLR	2.4 [1.9–3.7]	2.6 [1.8–3.7]	0.922	2.2 [1.8–3.0]	3.5 [2.6–4.0]	0.045	2.5 [1.9–3.8]	2.3 [1.7–3.6]	0.295
PLR	165.5 [129.3–241.0]	184.8 [130.7–271.1]	0.399	154.9 [117.6–206.9]	252.8 [179.9–309.2]	0.020	170.9 [131.7–246.3]	177.3 [126.8–230.0]	0.565

All nutritional, inflammatory, and body composition markers were assessed preoperatively. Values are medians [interquartile range]. p-values were calculated using the Mann–Whitney U test for comparisons between the discontinuation and no-discontinuation groups. Doublet therapy = CAPOX or FOLFOX; monotherapy = S-1, UFT/LV or capecitabine. BMI: body mass index; PMI: psoas muscle index; PMD: psoas muscle density; mIMAC: modified intramuscular adipose tissue content; GNRI: Geriatric Nutritional Risk Index; PNI: Prognostic Nutritional Index; HALP score: hemoglobin–albumin–lymphocyte–platelet score; NLR: neutrophil-to-lymphocyte ratio; PLR: platelet-to-lymphocyte ratio.
